# Obstetrics1Read before the Eleventh Annual Meeting of the Illinois Veterinary Medical and Surgical Association.

**Published:** 1902-07

**Authors:** James M. Reed

**Affiliations:** Mattoon, Ill.


### OBSTETRICS.[1]

By James M. Reed, V.S.,
MATTOON, ILL.

Obstetrics is that branch of veterinary medicine and surgery which treats of female ailments, delivering of the young, and treating diseases of the genital organs. Until we become familiar with and better understand the practical parts, conservativeness will prevent us from accepting as gospel truth many of the fine-spun, hair-splitting theories now advanced. But give us time to properly digest them, and we will move on with the advance guard of progress in this direction. We are not displaying ignorance when we acknowledge that in many instances it is a very difficult matter in treating cases of abortion to distinguish between it and some other form of pain which pertains to some other disease. In some cases great responsibility rests upon our diagnosis of the case. We know that grave mistakes have sometimes been made by which the school of practice has been closed. And we also know that where these mistakes have occurred people have lost confidence in the judgment and ability of the practitioner, and often dislike to pay the fees for his services. We cannot afford to be too decided in this matter. There are exceptions to all rules,

[1] Read before the Eleventh Annual Meeting of the Illinois Veterinary Medical and Surgical Association.

and a mode of treatment cannot be determined upon until the case is diagnosed. Communities are observant, and charge us with such shortcomings. Besides, they do not care whether we arrive at our conclusions through the medium of a culture test taken from the patient present, or through symptoms familiar to us by close attention to our profession in a long line of practical experience. Breeders of the domestic animals object to being deceived by the want of skilful or competent services. When placing their stock at our mercy we might be asked how we are to avoid making such mistakes when at times it is so difficult to discern a case of abortion from other pain-producing diseases. To which our only answer can be that "Experience and intimate knowledge of the actual thing itself can be our only guide." To the general practitioner that does not signify very much, for he knows that very few who may chance to witness such cases are ignorant of the real trouble. I sometimes think that all a veterinary surgeon requires to be able to remove the dead and completely rotted fœtus from the womb of an old cow which has been fed on young green clover, and to retain his shoe heels, is to have the digestive organs of an old turkey buzzard.

While, to be a successful practitioner, one must have some knowledge of obstetrics as laid down in our text-books, he must also have some practical knowledge as taught in every-day experience. I well remember how attentively I pondered over my text-books of the leading writers on veterinary medicine, the descriptions to the student therein given, and how we should go at the many presentations of the unborn. While I faithfully follow the principles of obstetrics as taught in our leading text-books, I find nothing therein telling me how to handle the female when she places herself in some of the many difficult positions for operation. Even while following the results of observations and conclusions of my theoretical knowledge and practical experiences and having exhausted all my resources, I have still made some wonderfully successful failures. It is true that in many instances I have had no trouble whatever. We know that many of our patients have unpleasant quarters, which aggravates the case and adds to our difficulties in treating it. Now, it is reasonable to suppose that others have had the same thing to contend with, and have had the same lack of success, for it is improbable that all of my cases were the only exceptions. One professional gentleman stated to me that his history of experience in abortions and delivering of the young was always a success to the mother, and, in a great many cases, the young were also saved, and that he had a certain line of treatment to which he always adhered. This may be true, but, in my opinion, his cases in obstetrics are few and far between. Such positive statements of absolute success in all cases always struck me as being very "fishy."

Propound a few of the leading questions to such a veterinarian, and you will soon lose his company; you will see that this kind of pressure puts him to flight, and, to hold on to him, you will have to be heavier than I am, or you will be dangling in the air like the tail end of a kite before a stiff breeze. In the practice of veterinary medicine I have met with many peculiar presentations. Now, suppose you have a case of muzzle presentation. After preparing yourself in the proper paraphernalia, and applying antiseptic precautions, you would introduce your hand and raise the nose. After which nature would, in all probability, do the rest, or possibly a little of your assistance might be required. Suppose you had a lumbosacral presentation: you would simply take it by the heels and politely pull the young fellow into the world. Should you have a case of posterior limbs and anterior presentation: I would, in my treatment of this case, reduce it to a lumbosacral presentation, which, to my mind, would be the safest to the mother, especially if it was a very large fœtus. I might mention many of these different presentations to which, my experience teaches me, no one rule or mode of treatment will apply, especially in cases where there have been several hours of continuous labor. The ideal treatment of such cases is that by which a perfect aseptic condition should be obtained and presented where this is possible. The object of this treatment becomes changed to the application of means to diminish the activity of the septic organism and to secure the rapid renewal of their production, and to increase the resisting powers of the wounded tissue. I do not know how to sufficiently emphasize the importance and actual necessity of cleanliness in each and every detail. Not much can be expected from practitioners with unkempt surroundings, and who, in their actual daily practice, belittle their calling by their disregard of water and careless toleration of dirt. From the natural order of things they cannot be expected to follow the scientific principles in this branch of veterinary medicine.

